# Endothelin 1-induced retinal ganglion cell death is largely mediated by JUN activation

**DOI:** 10.1038/s41419-020-02990-0

**Published:** 2020-09-26

**Authors:** Olivia J. Marola, Stephanie B. Syc-Mazurek, Gareth R. Howell, Richard T. Libby

**Affiliations:** 1 Department of Ophthalmology, Flaum Eye Institute, University of Rochester Medical Center, Rochester, NY USA; 2 Cell Biology of Disease Graduate Program, University of Rochester Medical Center, Rochester, NY USA; 3 The Center for Visual Sciences, University of Rochester, Rochester, NY USA; 4 Medical Scientist Training Program, University of Rochester Medical Center, Rochester, NY USA; 5 The Jackson Laboratory, 600 Main Street, Bar Harbor, ME USA; 6 Department of Biomedical Genetics, University of Rochester Medical Center, Rochester, NY USA

**Keywords:** Apoptosis, Mechanisms of disease, Neurodegeneration, Retina

## Abstract

Glaucoma is a neurodegenerative disease characterized by loss of retinal ganglion cells (RGCs), the output neurons of the retina. Multiple lines of evidence show the endothelin (EDN, also known as ET) system is important in glaucomatous neurodegeneration. To date, the molecular mechanisms within RGCs driving EDN-induced RGC death have not been clarified. The pro-apoptotic transcription factor JUN (the canonical target of JNK signaling) and the endoplasmic reticulum stress effector and transcription factor DNA damage inducible transcript 3 (DDIT3, also known as CHOP) have been shown to act downstream of EDN receptors. Previous studies demonstrated that JUN and DDIT3 were important regulators of RGC death after glaucoma-relevant injures. Here, we characterized EDN insult in vivo and investigated the role of JUN and DDIT3 in EDN-induced RGC death. To accomplish this, EDN1 ligand was intravitreally injected into the eyes of wildtype, Six3-cre^+^*Jun*^*fl/fl*^ (*Jun*^*−/−*^), *Ddit3* null (*Ddit3*^*−/−*^), and *Ddit3*^*−/−*^*Jun*^*−/−*^ mice. Intravitreal EDN1 was sufficient to drive RGC death in vivo. EDN1 insult caused JUN activation in RGCs, and deletion of *Jun* from the neural retina attenuated RGC death after EDN insult. However, deletion of *Ddit3* did not confer significant protection to RGCs after EDN1 insult. These results indicate that EDN caused RGC death via a JUN-dependent mechanism. In addition, EDN signaling is known to elicit potent vasoconstriction. JUN signaling was shown to drive neuronal death after ischemic insult. Therefore, the effects of intravitreal EDN1 on retinal vessel diameter and hypoxia were explored. Intravitreal EDN1 caused transient retinal vasoconstriction and regions of RGC and Müller glia hypoxia. Thus, it remains a possibility that EDN elicits a hypoxic insult to RGCs, causing apoptosis via JNK-JUN signaling. The importance of EDN-induced vasoconstriction and hypoxia in causing RGC death after EDN insult and in models of glaucoma requires further investigation.

## Introduction

Glaucoma is a neurodegenerative condition affecting the output neurons of the retina—the retinal ganglion cells (RGCs). The mechanisms that cause RGC insult and death in glaucoma remain incompletely understood. Recently, the endothelin (EDN, also known as ET) system has been implicated in glaucomatous neurodegeneration^1–6^. There are three secreted EDN peptide ligands (EDN1, EDN2, and EDN3) and two EDN membrane-bound receptors (EDNRA and EDNRB). EDN ligands are the most potent vasoactive peptides known, and the canonical role of EDN signaling is to mediate vasoconstriction^4,7,8^. Changes in blood flow have been documented in human^9–12^ and animal models^5,6^ of glaucoma, and it is hypothesized that these changes could be important factors in the development and progression of glaucoma. EDN ligands and receptors were found to be upregulated in models of acute^13,14^ and chronic ocular hypertension^1,5,6,15–17^. Importantly, molecular clustering analysis of transcriptional changes in ocular hypertensive mouse retinas and optic nerve heads revealed that upregulation of *Edn* ligands and receptors was among the first molecular changes in glaucoma pathology^1,5,6^. Levels of EDN were also found to be elevated in the aqueous humor and plasma of glaucoma patients^3,18–20^. Antagonism or knockout of EDN receptors lessened RGC loss in models of acute and chronic ocular hypertension^1,5^. In addition, EDN delivery was sufficient to drive RGC death in vivo^2,4–6,21^. Despite these findings, the cell death pathways responsible for eliciting RGC death upon EDN insult remain unknown. Understanding the molecular mechanisms controlling EDN-induced RGC death in glaucoma will provide insight into early, critical pathways of glaucomatous neurodegeneration and can identify potential therapeutic targets for neuroprotective glaucoma treatments.

JUN N-terminal kinase (JNK) signaling has been shown to be important in mediating glaucoma-relevant neurodegeneration^22–26^. JNK signaling is a mitogen activated protein kinase phosphorelay system where MAP3Ks phosphorylate and activate MAP2Ks (MKK4 and MKK7), which phosphorylate and activate MAPKs (JNK). JNK phosphorylates and activates its canonical target JUN, which can then act as a proapoptotic transcription factor by promoting the transcription of pro-death genes (e.g. *Bim*, *Faslg*, and *Bbc3*)^24,27,28^. *Jun* deficiency or loss of function was significantly protective to RGCs after chronic ocular hypertension^25^ and axonal injury^22,26^. Similarly, endoplasmic reticulum stress has been implicated in neurodegenerative diseases, including glaucoma^24,26,29^. Prolonged endoplasmic reticulum stress promotes translation of DNA-damage-inducible transcript 3 (DDIT3, also known as CHOP), which can act as a pro-apoptotic transcription factor. *Ddit3* deficiency was moderately protective to RGCs after axonal injury and ocular hypertension^26,29,30^. Dual deletion of *Jun* and *Ddit3* conferred additive, near complete, and long-term protection to RGCs following optic nerve crush^26^. Given the potential importance of EDN signaling in glaucomatous neurodegeneration, it is plausible that EDN causes RGC death via JUN and/or DDIT3 activation. Notably, both JUN and DDIT3 have been known to act downstream of EDN receptors. MAPKs (including JNK) and JUN were shown to be upregulated or activated in a variety of cell types, including cultured RGCs, after exposure to EDN^31–40^. Therefore, JUN may play a role in regulating RGC death after EDN insult. The EDN system has also been shown to activate the endoplasmic reticulum stress response^41,42^. Therefore, it is possible that JUN and/or DDIT3 are RGC-intrinsic regulators of EDN-induced RGC death, similar to after glaucoma-relevant injuries. Here, we characterized the time course and extent of EDN insult and critically test the importance of JUN and DDIT3 in RGC death following EDN insult in vivo.

In addition to the apoptotic RGC-autonomous mechanisms triggered by EDN signaling, the cell types that respond to EDN ligands to ultimately cause RGC death remain unknown. EDN ligands cause vasoconstriction by binding to EDNRA expressed by vascular smooth muscle cells^4,7,8,43,44^. Thinner retinal vessels and evidence of hypoxia have been demonstrated in animal models of chronic glaucoma^5,6,45,46^. Importantly, JUN was found be an important regulator of ischemia-induced neuronal death^47–51^. Thus, it is possible that EDN elicits a hypoxic insult to the retina via EDNRA to ultimately cause JUN-dependent RGC loss. To clarify how EDN ligands may be acting to cause RGC loss, we characterized changes in retinal vessel diameter and hypoxia after EDN insult.

## Materials and methods

### Mice

Wild-type (WT) C57BL/6J mice and mice carrying a germline deletion of *Ddit3*^52^ (The Jackson Laboratory, Stock# 005530) or floxed alleles of *Jun*^53^ (*Jun*^*fl*^) were utilized for all experiments. All mice used were 2–6 months of age. Floxed alleles were recombined in the optic cup using Six3-cre^54^ (The Jackson Laboratory, Stock# 019755). All alleles were backcrossed >10 times to the C57BL/6J genetic background (>99% C57BL/6J), and all colonies were maintained by intercrossing. Mice were fed chow and water ad libitum and housed on a 12-h light-to-dark cycle. Roughly equal numbers of males and females were used for each experimental group. Animals were randomly assigned to experimental groups. Before experiments were performed, it was established that animals with pre-existing abnormal eye phenotypes (e.g. displaced pupil, cataracts) would be excluded from the study. All experiments were conducted in adherence with the Association for Research in Vision and Ophthalmology’s statement on the use of animals in ophthalmic and vision research and were approved by the University of Rochester’s University Committee on Animal Resources.

### Intravitreal injections

Mice were anaesthetized with an intraperitoneal injection of 0.05 ml/10 g solution containing ketamine (20 mg/ mL) and xylazine (2 mg/ mL). Eyes were sterilized with 50% betadine solution made in 1X phosphate buffered saline (PBS), and were thoroughly flushed with 1X PBS. The conjunctiva at the temporal quadrant was cleared away with the bevel of a 30-gauge needle, and a small incision was made with the 30-gauge needle through the sclera and behind the limbus. Vitreous was allowed to drain, and any blood was wicked away. Five μL Hamilton syringes (Hamilton Company, 7633–01) with a blunt 33-gauge needle were used to perform intravitreal injections. The needle of the Hamilton syringe was inserted 1 mm into the incision site at a 45° angle toward the optic nerve, and was held in place for 30 s prior to injection. Care was taken to avoid contacting the lens with the Hamilton needle. Before experiments were performed, it was established that eyes with observable lens damage as a result of intravitreal injection would be excluded from the study. Over the course of 2 min (to prevent a sudden increase in intraocular pressure), 2 μL of 500 μM EDN1 (Sigma, E7764), diluted in sterile 1X PBS was injected into the vitreous. The Hamilton needle was held in place for 30 s following the injection, and the needle was removed from the eye over the course of 30 s. Antibiotic ointment was placed over the eye. The contralateral eye was injected with 2 μL of vehicle (sterile 1X PBS) with identical methods to serve as a control. Note: the investigator was masked to genotype while performing intravitreal injections.

### Fluorescein angiography

Fluorescein angiography was performed as previously described^55^. Mice were anaesthetized with an intraperitoneal injection of 0.05 mL/10 g solution containing ketamine (20 mg/mL) and xylazine (2 mg/mL). Mice were given an intraperitoneal injection of 0.2 μL/g fluorescein (25% Fluorescein Sodium) immediately prior to angiography. Retinal vasculature was photographed using a Micron III mouse retinal imaging system (Phoenix Research Labs).

### Hypoxia detection

Hypoxia was assessed using pimonidazole HCl (Hypoxyprobe) according to manufacturer’s instructions. Ninety minutes prior to euthanasia, mice were given an intraperitoneal injection of (60 mg/kg) pimonidazole HCl diluted in sterile 1X PBS. Mice were then transcardially perfused with 20 mL saline solution and 20 mL 4% paraformaldehyde. Eyes were harvested and incubated in 4% paraformaldehyde for 30 min. Eyes were stored in 1X PBS until dissection. Hypoxia was detected using an antibody against pimonidazole (Table 1).

**Table 1:** Summary of antibodies.

	Concentration	Company
Primary antibody (catalog number)		
Rabbit anti-cCASP3 (AF835)	1:1000	R&D Systems
Goat anti-CD31 (AF3628)	1:1000	R&D Systems
Goat anti-ChAT (AB144P)	1:200	Millipore
Mouse anti-JUN (BDB610327)	1:250	Fisher Scientific
Rabbit anti-pimonidazole (PAb2627AP)	1:200	Hypoxyprobe
Rabbit anti-pJUN (9261S)	1:250	Cell Signaling
Rabbit anti-RBPMS (GTX118619)	1:250	GeneTex
Guinea pig anti-RBPMS (1832-RBPMS)	1:250	PhosphoSolutions
Goat anti-SOX2 (sc-17320)	1:200	Santa Cruz
Secondary antibody (catalog number)		
Donkey anti-rabbit (A31572, A-21206)	1:1000	ThermoFisher
Donkey anti-guinea pig (706–605–148)	1:1000	Jackson ImmunoResearch
Donkey anti-goat (A21447)	1:1000	ThermoFisher
Donkey anti-mouse (A31570)	1:1000	ThermoFisher

### Immunostaining and quantification

Eyes were harvested and fixed in 4% paraformaldehyde in 1X PBS for 90 min. For retinal sections, the anterior segment was removed and the retina was kept in the optic cup for processing. Retinas were incubated in 10% sucrose (in 1X PBS) overnight, 20% sucrose overnight, and 30% sucrose for 3 days. Eyes were embedded in tissue freezing medium (General Data, TFM-5), frozen at −80 °C, and cross-sectioned at 14 μm. Sections were collected on glass microscope slides, which were stored at −20 °C. For hypoxia detection, one section every 84 μm was collected for immunofluorescence to ensure representation from the entire retina. Sections were blocked in 10% horse serum, 0.1% TritonX in 1X PBS for 3 h at room temperature and then incubated in primary antibody made in 10% horse serum, 0.1% TritonX overnight at 4 °C. Sections were washed in 1X PBS and incubated in secondary antibody made in 1X PBS for 2 h at room temperature. Sections were then washed, counterstained with 4′,6-diamidino-2-phenylindole (DAPI), and mounted in Fluorogel in TRIS buffer (Electron Microscopy Sciences). A masked observer captured images. For hypoxyprobe immunofluorescence, images were exposure-matched.

Methodology for cell quantification in retinal sections was adapted from Harder et al.^56^. All sections analyzed were a maximum of 168 μm from the optic nerve head. Five equidistant 20× fields (444 μm) were captured per section; two at each periphery, two at each middle area, and one at the center. Four sections were analyzed per retina. The cell counter tool in ImageJ was used for cell quantification. For each section, cell counts were summed. The score of each retina was the average of the 4 sections’ summed cell counts normalized to the distance measured.

For whole mount staining, retinas were dissected free from the optic cup and blocked in 10% horse serum, 0.4% TritonX in 1X PBS overnight at 4 °C. Retinas were incubated at 4 °C for 72 h in primary antibody diluted in 10% horse serum, 0.4% TritonX in 1X PBS. Retinas were then washed and incubated overnight at 4 °C in secondary antibody diluted in 1X PBS. Retinas were washed and mounted ganglion cell layer-up in Flourogel in TRIS buffer (Electron Microscopy Sciences). Note: for triple immunofluorescence, primary antibodies from three different host species were utilized, and secondary antibodies conjugated with flourophores with 3 different excitation/ emission frequencies were utilized.

As previously described^22–26^, RBPMS+, SOX2+, and pJUN+ cells were quantified using eight 40× fields per retina, and cCASP3+RBPMS+ cells were quantified using eight 20× fields per retina. Images were equally spaced 220 μm from the peripheral edge of the retina. The cell-counter tool in ImageJ was used for quantification. A masked observer captured and quantified images.

To assess changes in artery and vein diameter after EDN1 insult ex vivo, mice were trancardially perfused with 20 mL PBS and 20 mL 4% PFA 5 min after EDN1 injection. Retinas were processed for whole mount immunostaining as described above. Retinal artery and vein diameters were measured using the line segment measurement tool in ImageJ. Six measurements per CD31+ artery and vein were taken 240 μm, 305 μm, 370 μm, 435 μm, 500 μm, and 565 μm from the optic disc. Distance from the optic disc was chosen in order to include measurments before the approximate location of the first arterial bifurcations. Arteries and veins were identified by their distinct branching morphologies^57^.

### Controlled optic nerve crush

Controlled optic nerve crush (CONC) was performed as previously described^27,58^. Mice were anesthetized with an intraperitoneal injection of 0.05 ml/10 g solution containing ketamine (20 mg/mL) and xylazine (2 mg/mL). Meloxicam (2 mg/kg) was administered subcutaneously prior to surgery. The optic nerve was exposed and crushed immediately behind the eye with self-closing forceps for 5 s. Antibiotic ointment was applied to the eyes after the procedure.

### Statistical analysis

**Fig. 1:**
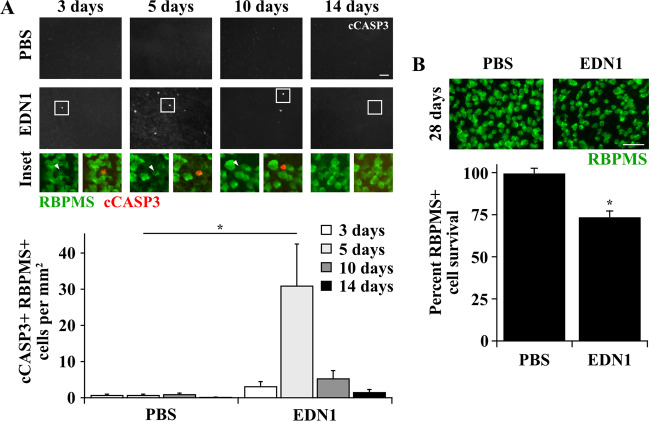
EDN1 caused caspase 3 activation in RGCs and RGC loss. **a** Caspase 3 activation in RBPMS+ RGCs after intravitreal PBS or EDN1 injection. Caspase 3 was activated (cleaved, cCASP3, red) in RBPMS+ RGCs (green) post-EDN1 injection, which peaked after 5 days. Caspase 3 was rarely activated in RGCs at any timepoint post-PBS injection (cCASP3+ RBPMS+ cells/mm^2^ ± SEM 3, 5, 10, 14 days post injection; PBS: 0.8 ± 0.2, 0.8 ± 0.2, 1.0 ± 0.3, 0.1 ± 0.1; EDN1: 3.2 ± 1.3, 31.0 ± 11.5, 5.4 ± 2.1, 5.4 ± 2.1, 1.6 ± 0.7; *n* ≥ 5 per condition per timepoint, *P < 0.001 compared to respective PBS, two-way ANOVA, Holm-Sidak’s post hoc). Note: bottom row images depict RBPMS immunofluorescence (left image, green) and RBPMS/cCASP3 merged immunofluorescence (right image, green and red respectively) from the boxed area of the above image. Arrowheads indicate an RBPMS+ cCASP3+ cell. **b** EDN1-injured retinas had significantly fewer RBPMS+ RGCs (green) compared to PBS controls 28 days post injection (%RBPMS+ cell survival ± SEM; PBS: 100.0 ± 2.6; EDN1: 73.9 ± 3.3; *n* ≥ 7 per condition, *P < 0.001, unpaired two-tailed *t* test). Note: in some EDN1-injured retinas, caspase activation and RGC death were localized to portions of retina and were not necessarily diffuse throughout the entire retina. Images represent the average cell count for each condition. Error bars, SEM. Scale bars, 50 μm.

**Fig. 2:**
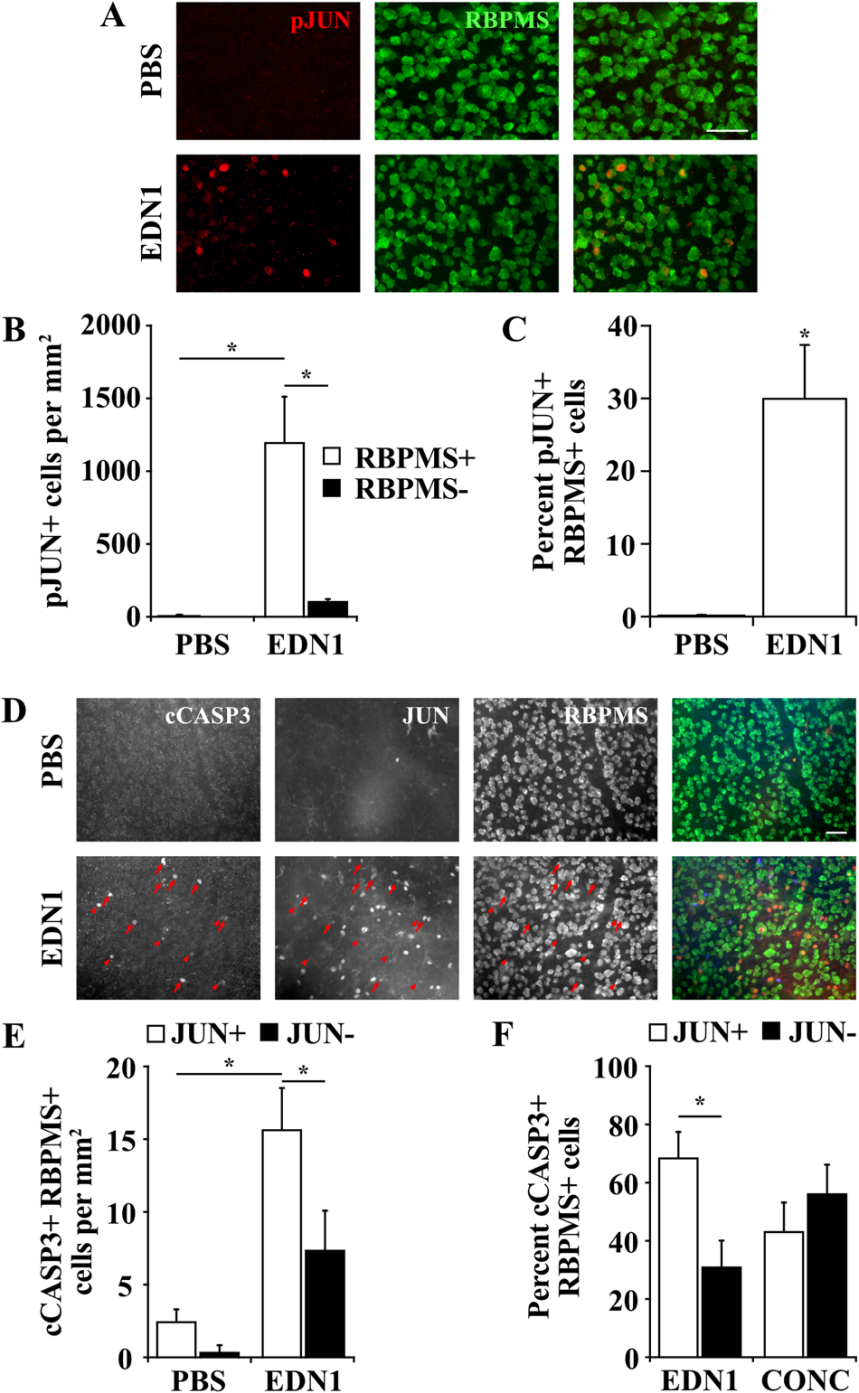
EDN1 caused JUN activation in RGCs. **a** Retinal flat mounts depicting JUN activation in RBPMS+ RGCs 24 h after PBS and EDN1 injection. **b** Quantification of RBPMS+ and RBPMS- pJUN+ cells 24 h after PBS and EDN1 injection. JUN was accumulated and activated (phosphorylated, pJUN, red) in 1203.8 ± 9.4 RBPMS+ cells per mm^2^ after EDN1 injection. A small portion of pJUN+ cells (113.0 ± 9.4 cells per mm^2^) were RBPMS−. *n* = 4 per condition, *P < 0.001, two-way ANOVA, Holm-Sidak’s post hoc. **c** Percentage of pJUN+ RBPMS+ cells after PBS and EDN1 injection. JUN was accumulated and activated (phosphorylated, pJUN, red) in a portion of RBPMS+ (green) RGCs 24 h post-EDN1, and was rarely activated in RBPMS+ RGCs post-PBS (%pJUN+ RBPMS+ cells; PBS: 0.1 ± 0.1; EDN1: 30.2 ± 7.2; *n* = 4 per condition, *P = 0.001, Welch’s *t* test). Note: in some EDN1-injured retinas, JUN activation in RGCs was localized to portions of retina and was not necessarily diffuse throughout the entire retina. Images represent the average cell count for each condition. **d** Retinal flat mounts depicting caspase 3 activation and JUN accumulation in RBPMS+ RGCs 5 days post- PBS and EDN1 injection. Arrows indicate cCASP3+ JUN+ cells, arrowheads indicate cCASP3+ JUN- cells. Merged images (far right) depict cCASP3+ (blue), JUN+ (red), and RBPMS+ (green) cells. **e** Quantification of cCASP3+ RBPMS+ cells that were JUN+ and JUN- after PBS and EDN1 injection. After PBS, 0.9 ± 0.1 cells/mm^2^ were RBPMS+ cCASP3+ JUN+, and 0.1 ± 0.1 cells/mm^2^ were RBPMS+ cCASP3+ JUN-. After EDN1, 15.7 ± 2.8 cells per mm^2^ were RBPMS+ cCASP3+ JUN+, and 7.4 ± 2.7 cells per mm^2^ were RBPMS+ cCASP3+ JUN−. (*n* = 6 per condition, *P < 0.05, two-way ANOVA with Holm-Sidak’s post hoc test). **f** Comparison of the percentage of cCASP3+ RBPMS+ cells that were JUN+ and JUN− between EDN1 injured retinas (*n* = 6) and controlled optic nerve crush-injured retinas (CONC; *n* = 4) 5 days post injury. Of all cCASP3+ RBPMS+ cells, 68.8 ± 8.7% were JUN+ after EDN1 insult, and 43.8 ± 9.6% were JUN+ after controlled optic nerve crush. (*P < 0.05, two-way ANOVA with Holm-Sidak’s post hoc). Scale bars, 50 μm.

**Fig. 3:**
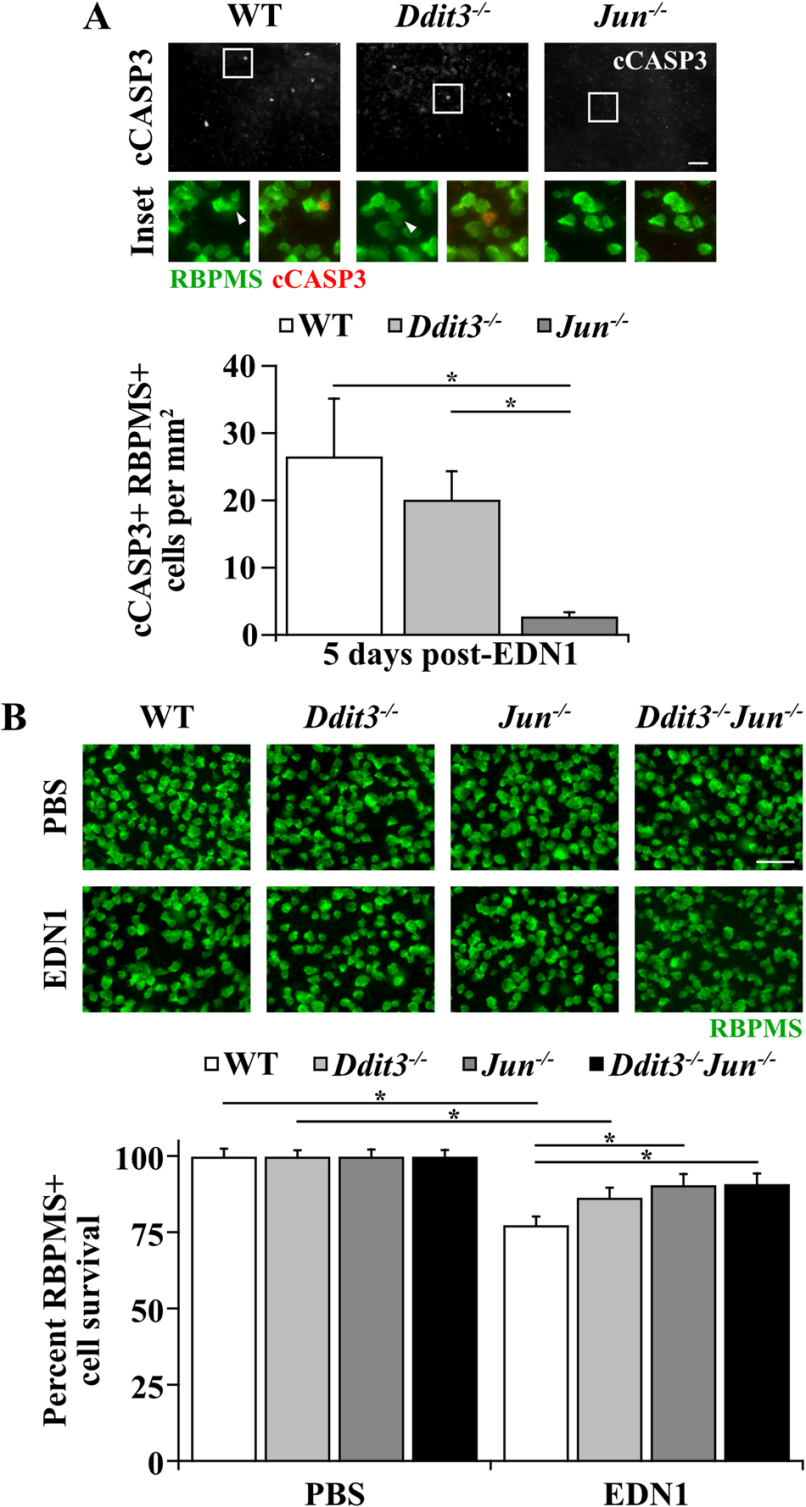
EDN1-induced RGC death was JUN-dependent. **a** Caspase 3 activation (cleavage, cCASP3, red) in RBPMS+ RGCs (green) 5 days post-EDN1 in WT, *Ddit3*^*−/−*^, and *Jun*^*−/−*^ RGCs. Caspase 3 activation was not significantly lessened in *Ddit3*^*−/−*^ RGCs compared to WT (*P* > 0.999), and *Jun* deletion from RGCs with Six3-cre significantly attenuated caspase 3 activation in RGCs compared to WT (*P < 0.001) and *Ddit3*^*−/−*^ retinas (*P = 0.004). cCASP3+ RGCs/mm^2^ ± SEM for WT, *Ddit3*^*−/−*^, *Jun*^*−/−*^, respectively: 26.6 ± 8.6; 20.1 ± 4.2; 2.8 ± 0.6; *n* ≥ 7 per genotype, *P < 0.001, Kruskal–Wallis test, Dunn’s post hoc. Note: bottom row images depict RBPMS immunofluorescence (left image, green) and RBPMS/cCASP3 merged immunofluorescence (right image, green and red respectively) from the boxed area of the above image. Arrowheads indicate an RBPMS+ cCASP3+ cell. **b** %RBPMS+ (green) RGC survival in WT, *Ddit3*^*−/−*^, *Jun*^*−/−*^, and *Ddit3*^*−/−*^*Jun*^*−/−*^ retinas 28 days post-PBS or EDN1. Both WT and *Ddit3*^*−/−*^ retinas had significant RGC loss 28 days post-EDN1 insult compared to corresponding PBS-injected controls, while *Jun*^*−/−*^ and *Ddit3*^*−/−*^*Jun*^*−/−*^ retinas did not have significant RGC loss (WT: *P < 0.001; *Ddit3*^*−/−*^: *P = 0.012; *Jun*^*−/−*^: *P* = 0.139; *Ddit3*^*−/−*^*Jun*^*−/−*^: *P* = 0.244). *Jun*^−/−^ retinas had significantly more surviving RGCs after EDN1 compared to WT (*P = 0.015). *Ddit3*^*−/−*^*Jun*^*−/−*^ RGCs were also significantly protected from EDN insult compared to WT (*P = 0.022), but *Ddit3*^*−/−*^*Jun*^*−/−*^ RGCs did not have significantly more protection than did *Jun*^*−/−*^ RGCs (*P* > 0.999). %RGC survival ± SEM for WT, *Ddit3*^*−/−*^*, Jun*^*−/−*^, *Ddit3*^*−/−*^*Jun*^*−/−*^, respectively: PBS: 100.0 ± 2.4, 100.0 ± 1.9, 100.0 ± 2.2, 100.0 ± 2.0; EDN1: 77.5 ± 2.7, 86.5 ± 3.2, 90.6 ± 3.5, 90.9 ± 3.3; *n* ≥ 12 per genotype per condition. Data were analyzed using a two-way ANOVA with Holm-Sidak’s post hoc test. Note: in some EDN1-injured retinas, caspase 3 activation in RGCs and RGC loss were localized to portions of retina and was not necessarily diffuse throughout the entire retina. Images represent the average cell count for each condition. Error bars, SEM. Scale bars, 50 μm.

**Fig. 4:**
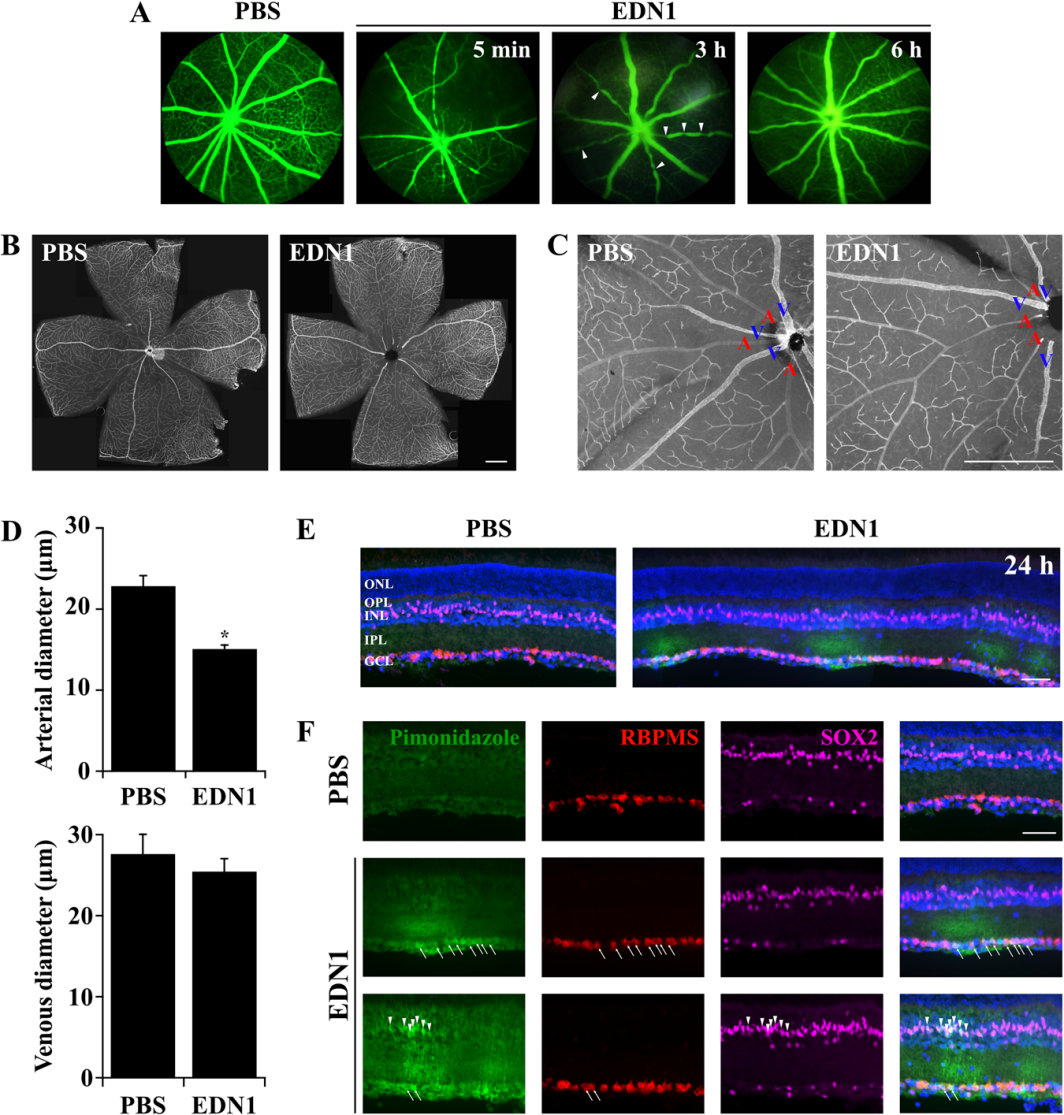
EDN1 induced vasoconstriction and RGC hypoxia. **a** Fluorescein angiography of retinal vessels after intravitreal PBS and EDN1 injections. EDN1 caused potent retinal vasoconstriction beginning rapidly after injection and lasting up to 3 h (arrowheads indicate areas of vasoconstriction at 3 h post-EDN1). Vessels appeared normally perfused by 6 h post injection (*n* ≥ 3 per condition per timepoint). **b** Stitched images of retinal flat mounts immunoassayed with CD31 5 min post-PBS and EDN1 injection. Scale bar, 500 μm. **c** Higher magnification of retinal flat mounts immunoassayed with CD31 5 min post-PBS and EDN1 injection. Veins are indicated with blue “V”s, arteries are indicated with red “A”s. After EDN1 injection, retinal arteries were markedly thinner compared to PBS. Scale bar, 500 μm. **d** Quantification of arterial and venous diameter 5 min after PBS and EDN1 injection. Retinal arteries, but not veins, were significantly thinner in diameter after EDN1 injection. Artery diameter (μm) ± SEM; PBS: 23.0 ± 1.2; EDN1: 15.2 ± 0.4, *n* = 6 per condition, *P < 0.001, Welch’s *t*-test. Vein diameter (μm)± SEM; PBS: 27.7 ± 2.4; EDN1: 25.6 ± 1.5, *n* = 6 per condition, *P* = 0.338, two-tailed *t* test. **e** Retinal sections depicting hypoxia (assessed with pimonidazole, green), RGCs (RBPMS, red), Müller glia (SOX2, magenta), and DAPI (blue) after intravitreal PBS and EDN1 (note: the EDN1-injured retina depicted in **e** is 3 stitched images). Regional hypoxia began as early as 3 h post-EDN1 insult (0/5 PBS exposed retinas and 5/5 EDN1 exposed retinas had pimonidazole + RGCs, data not shown), and continued out to 24 h post insult (0/5 PBS-exposed retinas and 5/5-EDN1 exposed retinas had pimonidazole + RGCs and Müller glia). Note: in EDN1-injured retinas, regions of hypoxia were often localized to portions of retina and were not necessarily diffuse throughout the entire retina. Pictured is an example of several hypoxic regions in an EDN1-injured retina. ONL outer nuclear layer; OPL outer plexiform layer; INL inner nuclear layer; IPL inner plexiform layer; GCL ganglion cell layer. Scale bar, 50 μm. **f** Individual channels depicting pimonidazole (green), RGCs (RBPMS, red), Müller glia (SOX2, magenta), and DAPI (blue) 24 h after PBS and EDN1. Arrows indicate pimonidazole + RGCs, arrowheads indicate pimonidazole+ Müller glia. Scale bar, 50 μm.

**Fig. 5:**
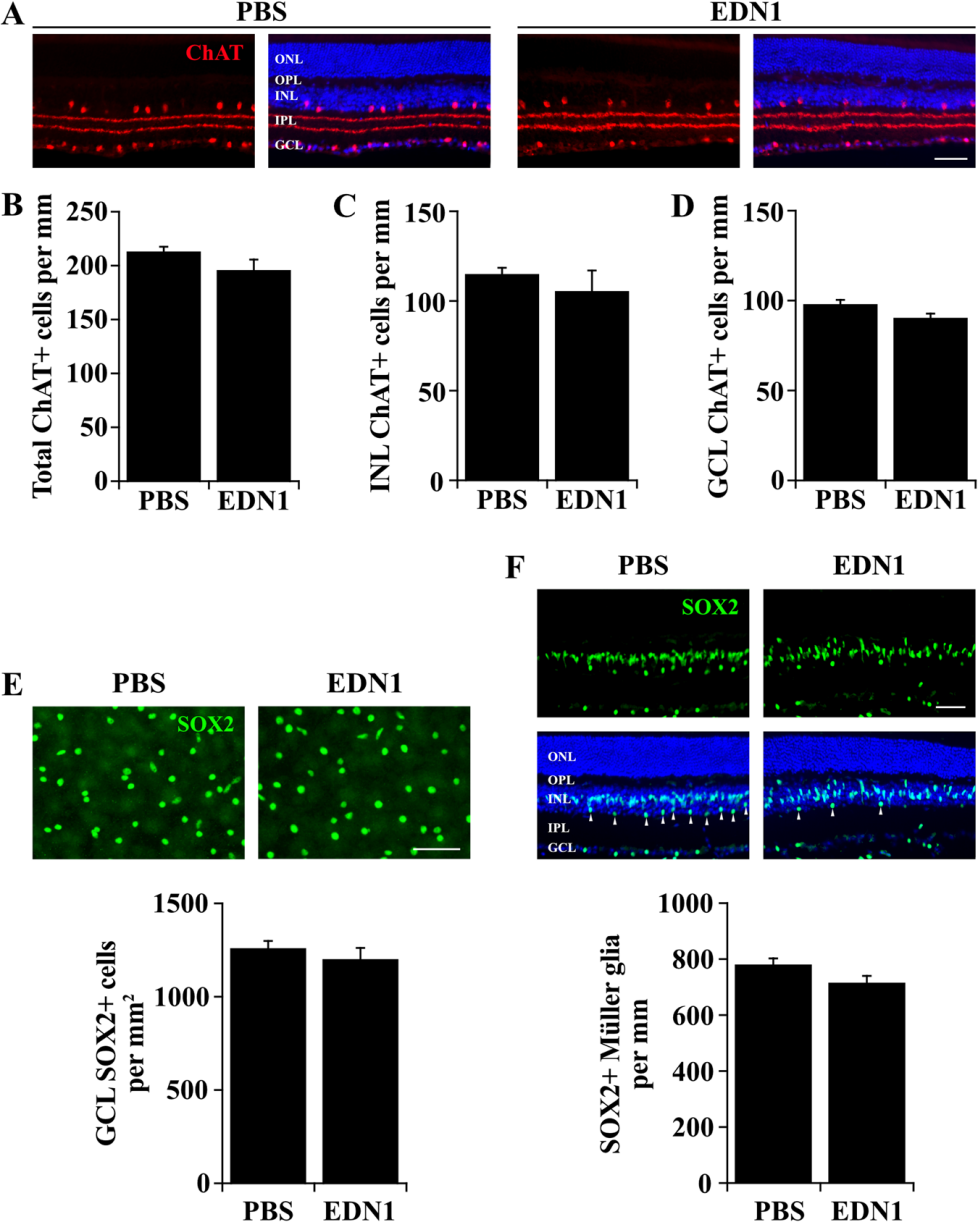
EDN1 did not cause loss of amacrine cells or Müller glia. **a** Retinal sections depicting choline acetyltransferase + (ChAT, red) amacrine cells and synaptic strata in the inner plexiform layer 28 days after PBS and EDN1 injection. Amacrine cell synaptic strata were morphologically indistinct 28 days after PBS and EDN1. **b** Quantification of total ChAT+ cells 28 days after PBS and EDN1. EDN1 injured retinas had similar numbers of ChAT+ amacrine cells compared to controls (ChAT+ cells/mm± SEM; PBS: 213.6 ± 4.0; EDN1: 196.4 ± 9.2, *n* ≥ 3 per condition, *P* = 0.117, two-tailed *t* test). ChAT+ amacrine cell numbers were similar in both the inner nuclear layer (INL; **c** INL ChAT+ cells/mm ± SEM; PBS: 115.3 ± 3.3; EDN1: 105.8 ± 11.3, *n* ≥ 3 per condition, *P* = 0.394, two-tailed *t* test) and the ganglion cell layer (GCL; **d**, GCL ChAT+ cells/mm± SEM; PBS: 98.3 ± 2.1; EDN1: 90.6 ± 2.2, *n* ≥ 3 per condition, *P* = 0.058, two-tailed *t* test). **e** Retinal flat mounts depicting SOX2+ amacrine cells in the ganglion cell layer 28 days after PBS and EDN1 injection. EDN1 did not cause significant loss of SOX2 + amacrine cells (SOX2 + cell survival ± SEM; PBS: 1263 ± 35.8; EDN1: 1204 ± 58.1; *n* = 7 per condition, *P* = 0.406, two-tailed *t* test). **f** Retinal sections depicting SOX2+ (green) Müller glia 28 days post-PBS and EDN1 injection. EDN1 did not appear to cause loss of SOX2+ Müller glia. Note: SOX2 labels a population of amacrine cells in the inner portion of the INL and in the GCL. Müller glia were distinguished from amacrine cells by location in the INL and by morphology in accordance with Surzenko et al.^81^. Arrowheads indicate SOX2+ amacrine cells in the INL that were not included in this quantification. (SOX2+ Müller glia/mm ± SEM; PBS: 783.1 ± 19.2; EDN1: 718 ± 22.0; *n* ≥ 3 per condition, *P* = 0.081, two-tailed *t* test). ONL outer nuclear layer; OPL outer plexiform layer; INL inner nuclear layer; IPL inner plexiform layer; GCL ganglion cell layer. Error bars, SEM. Scale bars, 50 μm.

Data were analyzed using GraphPad Prism8 software. Power calculations were performed before experiments were conducted to determine appropriate sample size. Data from experiments designed to test differences between two groups (e.g. %RGC survival, %pJUN+RBPMS+ cells, vessel diameters, ChAT+cells/mm, SOX2+ cells/mm^2^, and SOX2+ Müller glia/mm after PBS and EDN1 in WT mice; Figs. 1b, 2c, 4d, and 5) were subjected to an *F* test to compare variance and a Shapiro–Wilk test to test normality to ensure appropriate statistical tests were utilized. %RGC survival (Fig. 1b), venous diameter (Fig. 4d), ChAT+ cells/mm (Fig. 5b–d), SOX2+ cells/mm^2^ (Fig. 5e), and SOX2+ Müller glia/mm (Fig. 5f) were analyzed using unpaired two-tailed *t* tests. %pJUN+ RGCs (Fig. 2c) and arterial diameter (Fig. 4d) were analyzed using Welch’s *t* test. Data from experiments designed to test differences among more than two groups across one condition (e.g. caspase 3 activation among WT, *Ddit3*^*−/−*^, and *Jun*^*−/−*^ retinas after EDN1, Fig. 3a) were subjected to a Brown-Forsythe test to compare variance and a Shapiro–Wilk test to test normality to ensure an appropriate statistical test was utilized. Data were analyzed using a Kruskal–Wallis test with Dunn’s post hoc test. Data from experiments designed to detect differences among multiple groups and across multiple conditions (e.g. caspase 3 activation after PBS and EDN1 at multiple timepoints, RBPMS+ and RBPMS- pJUN+ cells after PBS and EDN1, pJUN+ and pJUN- cCASP3+ RBPMS+ cells after PBS and EDN1, %pJUN+ and %pJUN- cCASP3+ RBPMS+ cells after EDN1 and CONC, and %RGC survival across multiple genotype groups after PBS and EDN1; Figs. 1a, 2b, e, f, and 3b) were analyzed using a two-way ANOVA followed by Holm-Sidak’s post hoc test. For these statistical tests, every possible comparison was made when relevant, and multiplicity adjusted *P* values are reported. In all cases, data met the assumptions of the statistical test used. *P* values < 0.05 were considered statistically significant. Throughout the manuscript, results are reported as mean ± standard error of the mean (SEM).

## Results

### EDN1 caused caspase 3 activation in RGCs and RGC loss

Experiments using in vivo approaches have demonstrated that EDN exposure causes RGC death^4,6,21^. However, the mechanisms responsible for eliciting apoptosis upon EDN insult remain unknown. To understand the mechanisms of glaucoma-relevant EDN-induced RGC death, the time course and extent of EDN injury were characterized in vivo. To accomplish this, wild-type (WT) C57BL/6J mice were intravitreally injected with 2 μL of 500 μM EDN1 diluted in 1X PBS (1 nmol EDN1). 1X PBS was intravitreally injected into the contralateral eye to serve as an internal vehicle control. Note, this injection paradigm was used for all experiments. In models of chronic ocular hypertension, RGCs die via apoptosis^58^, a process mediated by cleavage of caspase 3. Therefore, retinas were immunoassayed for activated caspase 3 (cleaved caspase 3; cCASP3) at 3, 5, 10, and 14 days post-EDN1 and PBS injection. Retinas were counterstained for RBPMS, an RGC-specific marker^59^, to ensure caspase 3 activation was specific to RGCs. Intravitreal EDN1 caused caspase 3 activation in RBPMS+ RGCs; this activation peaked 5 days post injection and was sparse by 14 days post injection. cCASP3+ RGCs were rarely present at any timepoint post-PBS injection (Fig. 1a). Based on the time course of caspase 3 activation in RGCs, the extent of RGC death was assessed 28 days post-EDN1. At this timepoint, there was a 26% decrease in RBPMS+ RGCs compared to PBS controls (Fig. 1b). Thus, intravitreal EDN1 was sufficient to cause a significant loss of RGCs by 28 days after insult.

### EDN1 caused JUN activation in RGCs

The apoptotic mechanisms responsible for eliciting RGC death remain incompletely defined. JUN was shown to be important in RGC death after glaucoma-relevant insult^22,25,26^, and EDN exposure caused JUN upregulation in cultured primary RGCs^33^. To determine whether JUN plays a role in RGC death after EDN insult in vivo, retinas were immunoassayed for accumulated and activated (phosphorylated) JUN and RBPMS 1 day following PBS or EDN1 injection (prior to the onset of caspase 3 activation in RGCs, Fig. 1a). JUN accumulated and was activated (phosphorylated, pJUN) in 30% of RGCs 1 day following EDN1 injection (Fig. 2a–c), similar to the percentage of RGC death 28 days post-EDN injury (Fig. 1b). Of all pJUN+ cells, the vast majority (91.4%) were RBPMS+ RGCs, and a small population labeled an unknown cell type (Fig. 2b). JUN remained accumulated in RGCs 5 days post-EDN1 insult (Fig. 2d–f). To determine whether JUN+ RGCs were eventually the population of RGCs that apoptosed, retinas were immunoassayed for both JUN and cCASP3 5 days post-EDN1 insult. Of all cCASP3+ RGCs, 69% were also JUN positive (Fig. 2e). As a control, cCASP3+ RBPMS+ JUN+ cells were quantified after controlled optic nerve crush (CONC); an injury shown to cause RGC death via JUN signaling^26^. Similarly, only a portion (44%) of cCASP3+ RBPMS+ cells were also JUN+ (Fig. 2f). Given JUN accumulates in 100% of RGCs 1 day after CONC^26^, and only a portion of cCASP3+ RGCs are also JUN+ 5 days after CONC, it is likely that at this timepoint, cCASP3+ cells are in the process of degenerating, and proteins such as JUN are downregulated. Thus, JUN could feasibly contribute to EDN1-induced RGC death.

### EDN1-induced RGC death was largely JUN-dependent

The cell death pathways governing RGC death after EDN insult are not well defined. Considering EDN insult caused activation of JUN in 30% of RGCs (Fig. 2a–c) and 69% of cCASP3+ RGCs were also JUN+ after EDN1 insult (Fig. 2d–f), it remained possible that JUN regulates EDN1-induced RGC death, similar to glaucoma-relevant RGC death. To determine whether EDN1-induced RGC death occurs via a JUN-dependent mechanism, Six3-cre^+^*Jun*^*fl/fl*^ (herein referred to as *Jun*^*−/−*^) and WT (Six3-cre^+^*Jun*^*+/+*^; Six3-cre^−^*Jun*^*?*^) controls were intravitreally injected with EDN1 or PBS, and retinas were assessed for apoptotic RGCs and subsequent RGC loss. *Jun* deletion significantly lessened caspase 3 activation in RGCs 5 days post insult (Fig. 3a). Furthermore, retinal *Jun* deletion significantly attenuated RGC loss by 58% 28 days post insult (Fig. 3b).

Notably, *Jun* deletion did not protect all RGCs from EDN1-induced RGC death. This could be due in part to incomplete recombination of *Jun*^*fl*^ alleles by Six3-cre. Six3-cre recombined *Jun*^*fl*^ alleles in 80% of RGCs^26^. Given *Jun* deletion by Six3-cre protected fewer than 80% of RGCs after EDN1 insult, it is likely that another apoptotic mechanism(s) is important in mediating EDN1-induced RGC death. DDIT3 (also known as CHOP), a pro-apoptotic transcription factor activated after prolonged endoplasmic reticulum stress, moderately contributed to RGC death after glaucoma-relevant insults^26,29,30,60^. Importantly, previous studies have demonstrated that JUN and DDIT3 additively contributed to RGC death after axonal injury (*Ddit3* and *Jun* dual deletion prevented 75% of RGC death after RGC axonal insult)^26^. Therefore, we tested the effect of *Ddit3* deletion alone and in combination with *Jun* deletion on RGC survival after EDN insult. To accomplish this, mice with a homozygous germline deletion of *Ddit3* (*Ddit3*^*−/−*^) and WT littermate controls were utilized. *Ddit3* deletion did not significantly reduce caspase 3 activation 5 days post-EDN1 compared to WT controls, and *Ddit3* deletion did not significantly prevent RGC death 28 days post-EDN insult. To determine whether DDIT3 and JUN act in tandem to drive RGC death after EDN insult, *Ddit3*^*−/−*^*Jun*^*−/−*^ eyes were intravitreally injected with EDN1 or PBS. Dual deletion of *Ddit3* and *Jun* was significantly protective to RGCs 28 days post injection, but this was not significantly different than the effect of *Jun* deletion alone. Therefore, EDN1-induced RGC loss was largely JUN-dependent.

### EDN1 induced vasoconstriction and RGC hypoxia

The mechanisms by which EDN1 induces JUN signaling and ultimately apoptosis remains unclear. The canonical role of the EDN system is to mediate vasoconstriction. EDN ligands are the most potent vasoactive peptides, and EDN has been shown to cause vasoconstriction through EDNRA expressed by vascular smooth muscle cells^4,7,8,43,44^. Importantly, JUN-JNK signaling has been shown to mediate neuronal death after ischemic insult^47–51^. Therefore, it is possible that EDN1 elicits a hypoxic insult to the retina, which can ultimately lead to RGC death via JNK-JUN signaling. To determine whether EDN1 elicited retinal vasoconstriction, fluorescein angiography was utilized at several timepoints post-intravitreal injection of either EDN1 or PBS. As previously reported in rats^61–63^ and rabbits^64,65^, intravitreal EDN1 caused rapid retinal vasoconstriction, which lasted at least 3 h post injection. Retinal vessels appeared normally reperfused after 6 h. No vascular changes occurred at any timepoint post-PBS (Fig. 4a). Retinal vessel constriction was confirmed ex vivo 5 min post injection. EDN1 caused significant reduction of arterial diameter (Fig. 4b–d). EDN1 did not change retinal venous diameter. This is not surprising, as EDN ligands cause vasoconstriction by binding to EDNRA expressed by vascular smooth muscle cells, which are abundant in arterial walls but not in veins and therefore elicit more potent vasoconstriction of arteries^66,67^. Because vasoconstriction restricts blood flow, pimonidazole HCl (Hypoxyprobe) was utilized to determine whether intravitreal EDN1 caused retinal hypoxia. Pimonidazole forms covalent adducts in cells with a partial pressure of oxygen <10 mmHg. EDN1-injured retinas had hypoxic RBPMS+ RGCs and Müller glia somas (labeled by SOX2) and processes beginning 3 h post injection (data not shown) and lasting at least out to 24 h (Fig. 4e, f), suggesting that EDN1-induced vasoconstriction caused a hypoxic insult in the retina.

### EDN1 did not cause loss of amacrine cells or Müller glia

Chronic hypoxia has been shown to cause loss of retinal neurons; including RGCs and amacrine cells^68–71^. However, in glaucoma, neurodegeneration is specific to RGCs and does not affect other retinal neurons, such as amacrine cells^72^. Therefore, it was important to characterize whether EDN1 insult affects RGCs specifically, or whether EDN1 injures amacrine cells as well, potentially via a global retinal hypoxic insult. Therefore, choline acetyltransferase (ChAT) immunostaining was utilized to assess loss of amacrine cells and disruption of synaptic strata in the inner plexiform layer after EDN1 insult. After 28 days, EDN1 did not cause loss of ChAT+ amacrine cells (in the inner nuclear layer or the ganglion cell layer) or appear to disrupt the synaptic layers of the inner plexiform layer (Fig. 5a–d). Because approximately 59% of neurons in the ganglion cell layer are displaced amacrine cells^73^, SOX2+ displaced amacrine cell counts were assessed in the ganglion cell layer following EDN insult. SOX2 labels starburst amacrine cells in the ganglion cell layer^74^, which is by far the most abundant displaced amacrine cell. Therefore, SOX2+ cells in the ganglion cell layer were used to examine the effect of EDN1 insult on amacrine cells. There was no significant difference in the number of SOX2+ displaced amacrine cells in the ganglion cell layer between EDN1 and PBS injected eyes 28 days after injection (Fig. 5e). Therefore, similar to glaucomatous neurodegeneration, EDN1 insult affected RGCs and did not appear to cause amacrine cell loss. In addition, because hypoxyprobe labeling was observed in SOX2+ Müller glia after EDN1 insult, it was important to determine whether EDN1 insult elicited loss of Müller glia in addition to RGCs. After 28 days, EDN1 did not cause signicant loss of SOX2+ Müller glia (Fig. 5f). Therefore, EDN1 did not appear to cause a global retinal hypoxic insult.

## Discussion

Endothelin has been implicated as a mediator of glaucomatous RGC death^1,2,4–6,13,14,34,75,76^. Components of EDN signaling were upregulated in human^3,18–20^ and animal models of glaucoma^1,5,6,14–17^, and genetic deletion or antagonism of EDN receptors lessened glaucomatous damage in ocular hypertensive rodents^5^. Several groups have demonstrated EDN’s capacity to kill RGCs^2,4–6,21^. However, the mechanisms important in driving RGC death after EDN insult remain unidentified. Understanding the cell death pathways important in EDN-induced RGC death will further elucidate the earliest molecular mechanisms of glaucoma pathology and can potentially identify therapeutic targets for neuroprotection. In the present work, EDN1 insult to RGCs was characterized in vivo, and the roles of *Ddit3* and *Jun* in eliciting RGC death after EDN1 insult were assessed.

The cell-intrinsic mechanisms of EDN-induced RGC death remain incompletely understood. Given the relevance of EDN signaling to glaucomatous neurodegeneration, it is possible that EDN elicits RGC death via cell death pathways known to govern RGC death after glaucoma-relevant insult. MAPK-JNK signaling and its canonical target JUN regulated RGC death after glaucoma-relevant insults such as axonal injury^22,26^ and chronic ocular hypertension^25^. Similarly, but to a lesser extent, the endoplasmic reticulum stress effector and transcription factor DDIT3 played a role in RGC death after axonal injury^26^ and ocular hypertension^30^. Dual deletion of *Jun* and *Ddit3* conferred near-complete protection to RGCs after axonal insult^26^. MAPKs-including JNK- and JUN have been shown to act downstream of EDN receptors in multiple cell types^31,77,78^, including RGCs^33^. In addition, EDN receptors have been shown to potentiate the endoplasmic reticulum stress response, and *Ddit3* has been shown to act downstream of EDN receptors. Therefore, it remained possible that JUN and/or DDIT3 mediate RGC death in response to EDN.

In the present study, JUN was activated in 30% of RGCs 1 day post-EDN1 (Fig. 2a–c), and *Jun* deletion significantly attenuated RGC caspase 3 activation and RGC loss (Fig. 3a, b). This protection (58% increased RGC survival) is approximately commensurate to levels of protection conferred by *Jun* deletion in models of axonal injury (48% protection 120 days post injury)^26^ and chronic ocular hypertension (47% protection)^25^. In contrast, we found *Ddit3* deletion did not provide significant protection from EDN-induced RGC death. These data do not preclude the possibility that *Ddit3* deletion protected a small portion of RGCs after EDN insult that was not detected with our experimental design. This possibility is not unlikely, as *Ddit3* deletion protected only 25% of RGCs 120 days post-axonal insult^26^ and 20% of RGCs in ocular hypertensive mice^30^. Nevertheless, these data suggest that DDIT3 did not govern the majority of RGC death following EDN insult. Future work should elucidate the upstream regulators of JUN activation and the downstream targets of JUN signaling to further clarify the molecular mechanisms governing RGC apoptosis in response to EDN signaling. For instance, MAP3K12 (DLK)^23,26^, MAP2Ks 4 and 7 (MKK4, MKK7)^79^, and MAPKs 9 and 10 (JNK2, JNK3)^22^ are upstream regulators of JUN activation and were shown to be important in mediating glaucoma-relevant RGC death, and are perhaps important in regulating EDN-induced RGC death.

Furthermore, it will be important to determine whether *Jun* deletion protects RGC axons as well as somas after EDN insult. JUN has been shown to regulate RGC somal degeneration, but not axonal degeneration, after RGC axonal injury and chronic ocular hypertension. Theoretically, if EDN1 injures RGCs at the level of the soma, *Jun* deletion would likely confer protection to RGC axons as well as somas. However, if EDN1 causes a primary injury to RGC axons, *Jun* deletion could potentially confer protection to RGC somas, but not axons. Further dissecting the role of *Jun* in the degeneration of RGC somas and axons will be important for understanding EDN1-induced RGC death, and can possibly classify the location of EDN1 insult to RGCs (somal or axonal).

In addition to the cell death pathways important in eliciting RGC death after EDN insult, the retinal cell types that respond to EDN ligand and ultimately cause RGC death remain unknown. A multitude of cell types express EDN receptors; retinal neurons (including RGCs) and macroglia are known to express EDNRB, while RGCs and retinal mural cells are known to express EDNRA. While it is possible that EDN ligands affect RGCs directly (via receptors expressed by RGCs), it is also possible that EDN ligands signal extrinsically to RGCs by affecting glia or vascular cells and eliciting neurotoxic downstream effects; ultimately causing JUN-mediated RGC death. Regardless of the primary cell type EDN1 acts upon to ultimately cause RGC death, the present study indicates that ultimately, JUN is important in RGC degeneration after EDN1 exposure.

The canonical role of EDN signaling is to mediate vasoconstriction^4,7,8,43^. EDN ligands bind to EDNRA expressed by vascular mural cells, which elicits contraction of vascular smooth muscle cells and consequential vasoconstriction^4,7,8,43,44^. Thinner retinal vessels and reduced blood flow have been reported in human^9–12,80^ and animal models of glaucoma^5,6^. Notably, JNK-JUN signaling was found to contribute to neuronal death after ischemic insult to the rodent brain^47–50^ and retina^51^. Therefore, it is possible that aberrant EDN signaling via mural cell-EDNRA contributes to reduced retinal blood flow in glaucoma, which could in turn perpetuate neurodegeneration via JNK-JUN signaling.

Consistent with previous reports using rats^61–63^ and rabbits^64,65^, we found intravitreal EDN1 caused potent but transient arterial constriction in the mouse retina, which reperfused by 6 h post injection (Fig. 4a–d). Subsequently, EDN1 insult resulted in regions of hypoxic RGCs and Müller glia beginning 3 h (data not shown) and lasting at least 24 h post injection (Fig. 4b, c). Hypoxic glia and RGCs were also reported after induction of ocular hypertension, suggesting the potential importance of hypoxia in glaucoma pathology^45,46^. RGC death did not necessarily occur in close proximity to major vessels after EDN1 insult, but it is possible that loss of blood flow or damage to minor vessels and capillary beds contributes to EDN pathology. Future studies should investigate the effect of EDN on retinal vessel integrity, and how any changes may play a role in EDN-induced RGC death. Severe hypoxia is known to cause RGC and amacrine cell loss, however, we observed RGC loss (Fig. 1b) but not loss of ChAT+ or SOX2+ amacrine cells after EDN1 insult (Fig. 5a–e); consistent with glaucomatous neurodegeneration. In addition, although Müller glia hypoxia was observed 24 h after EDN1 insult, Müller glia survival appeared unaffected 28 days after EDN1. Thus, in the present study, EDN1-induced cell death appeared to be specific to RGCs, similar to glaucoma pathology, and it remains possible that hypoxia plays a role in glaucomatous and EDN-induced RGC death. Future work should elucidate the role of EDNRA and hypoxia in mediating RGC death after EDN1 insult and in models of glaucoma.
